# The global burden of chronic and hidden hunger revisited: New panel data evidence spanning 1990–2017

**DOI:** 10.1016/j.gfs.2020.100480

**Published:** 2021-03

**Authors:** Bert Lenaerts, Matty Demont

**Affiliations:** a UHasselt, Centre for Environmental Sciences, Hasselt, Belgium; b International Rice Research Institute (IRRI), Los Baños, Laguna, Philippines

**Keywords:** Hunger, Replication, Spatial spillovers, Cereals, Economic geography, DALYs

## Abstract

Gödecke, Stein and Qaim (2018) (GSQ) recently analysed the 1990–2010 trend and determinants of the global burden of chronic and hidden hunger. We reanalyse and extend GSQ's study and demonstrate that after 2010, significant reductions in the burden of hunger were achieved. In contrast with GSQ, we find that (i) hidden hunger is more prevalent than chronic hunger; (ii) cereal availability and the supply of vegetables and fruits matter; and (iii) gender equality only affects hidden hunger. We further provide evidence on the importance of spatial spillovers in GDP affecting the burden of hunger. Policy makers should therefore prioritize (i) enhancement of micronutrient density of cereals; (ii) diversification of production systems and consumer diets; and (iii) development of nutrition-sensitive food value chains.

## Highlights

• We reanalyse and extend the study of Gödecke et al. (2018).
• Hidden hunger is more prevalent than chronic hunger.
• Supply of cereals, vegetables and fruits significantly alleviates the burden of hunger.
• Gender equality only affects hidden hunger and not chronic hunger.
• Neighbours' GDP has a major positive effect on the alleviation of the burden of hunger.

## 1 Introduction

Policymakers face two non-exclusive options to address the burden of hunger: (i) tackle it directly through food security policies; or (ii) counteract its fundamental causes through policies on trade and transport connectivity to overcome geographic disadvantage (Gallup et al., 1999) or policies on agricultural production to overcome unproductive production environments (Beddington, 2010). However, they face two obstacles towards evaluating the global state of hunger and its determinants: (i) lack of country-level data; and (ii) reliance on proxies that only capture selected dimensions of hunger. Gödecke, Stein and Qaim (2018, 2019) (hereafter referred to as GSQ) overcome these problems by employing Disability-Adjusted Life Years (DALYs) from the first generation of the Global Burden of Disease (GBD) dataset quantifying the health burden of hunger in three discrete points in time, that is the years 1990, 2005 and 2010. The authors ﬁnd that (i) between 1990 and 2010, the total burden of chronic and hidden hunger diminished by more than 50 per cent and about 30 per cent, respectively; (ii) in 2010, the global burden of chronic hunger was larger than the global burden of hidden hunger; and (iii) economic growth, urbanisation, democracy, temperate-zone climates, larger food supplies, food diversity, female schooling, and access to improved sanitation and health care have contributed significantly to reducing the burden of chronic and hidden hunger. Remarkably, GSQ found no strong significant effect of cereal availability and the supply of vegetables and fruits on the global burden of chronic and hidden hunger, a result that warrants further investigation. Moreover, while GSQ included determinants of economic performance, demography, institutions and climate, their analysis did not include economic geography, which is an essential determinant of economic development and global food security (Fujita et al., 1999; Gallup et al., 1999).

Since the publication by GSQ, the fifth generation of DALY estimates has been released, which are calculated using an updated methodology and span a much broader timeframe (Global Burden of Disease Study 2017) (GBD 2017 GBD 2017 collaborators, 2018, 2019). To generate new evidence on the trends and determinants of the burden of hunger, we revisit the study of GSQ by using new panel data evidence for the period 1990–2017—as an extension and refinement of GSQ's three-point time estimates for 1990, 2005 and 2010—and we enrich the empirical analysis with new variables and alternative model specifications. First, we argue against the use of country-fixed effects panel models given that most factors related to the burden of hunger are fairly time-invariant. Instead, we propose a time-fixed model using two-way clustered standard errors. Secondly, we add determinants of economic geography and finally, we assess how the results are affected by subsampling those countries that are substantially affected by hunger.

## 2 Global food security and economic development

The global state of food security is largely a reflection of the global state of economic development (FAO et al., 2019). Indeed, global economic development is heterogeneous, as is global food security. Two centuries after the start of the First Industrial Revolution, economic development remains unevenly distributed, with sharp and diverging differences in average income between Western Europe and its offshoots, and the rest of the world (Bolt et al., 2018). While the prevalence of undernourishment in the world has been steadily declining, the total number of undernourished people in Africa, Western Asia and Oceania is larger now than it was a decade ago (FAO et al., 2019). A long-standing question in the literature is how to explain these regional differences in development (Bhattacharyya, 2016). This is important as food security is an outcome of economic development. Traditional economic models emphasise the importance of technological progress (Solow-Swan model; Solow, 1956; Swan, 1956) and the level of savings and capital accumulation (Harrod-Domar model; Ackley, 1961; Baumol, 1970). However, these models are problematic because their drivers are largely endogenous (being outcomes of the development process) and therefore need explanation themselves.

Acemoglu (2009) suggests three, non-mutually exclusive, fundamental causes of development: (i) geography, (ii) culture and (iii) institutions and policies. In this paper, we focus mainly on factors related to geography and governance. Geography can be further decomposed into (i) physical geography, (ii) biography, (iii) ecology and (iv) natural resource endowments. Physical geography such as centrality (Gallup et al., 1999), topography (Nunn and Puga, 2012) and landlockedness (Bloom et al., 1998; Demont, 2013; Faye et al., 2004; Gallup et al., 1999; Soullier et al., 2020) determine a country's natural level of market potential. This endowed level of market potential can be countered by a country's level of connectivity generated by advances in transport infrastructure and services (Lenaerts et al., in press; Pirie, 1993; Sheng et al., 2018) and trade facilitation (Brooks and Matthews, 2015; Dithmer and Abdulai, 2017; Matthews, 2014). Both features are an essential part of the concept of market access (Henderson et al., 2001; Lenaerts et al., 2020) underlying new economic geography theory (Krugman, 1991). The biogeographic hypothesis states that a large pool of wild plant and animal species suitable for domestication and a continental axis that is oriented east-west (permitting similar ecological zones) have shaped the history of human development in favour of Eurasia (Diamond, 1997). Ecology is understood as the living and production environment, which are characterised by factors such as temperature, rainfall, soil conditions, fragility to degradation and prevalence of pests and diseases (for both plants and animals, including humans) (Diamond, 2005; Gallup et al., 1999; Masters and McMillan, 2001; Nordhaus, 2006; Sachs, 2003). These factors both influence the inputs into the production function as well as the production function itself (Easterly and Levine, 2003). Unfavourable ecological conditions for agriculture can be compensated by advancements in agricultural technology (Beddington, 2010; Lenaerts et al., 2019). Lastly, natural resources endowments such as oil or metal reserves can, to a certain extent, determine a country's prosperity (Cavalcanti et al., 2011).

Second, cultural factors such as ethnolinguistic fragmentation can affect development, for example through imposing a coordination cost on countries, potentially leading to a negative impact on the quality of government and economic growth (Easterly and Levine, 1997; World Bank, 2008, p. 104).

Third, institutions and policies can play an important role in fostering development (Acemoglu and Robinson, 2012; Glaeser et al., 2004; Rodrik et al., 2004). As indicated before, good policies can (partially) counteract the forces behind environmental and cultural determinism. The relationship between institutions, geography and culture is more complicated. Extractive institutions might be historically related to ecology (Acemoglu et al., 2001; Easterly and Levine, 2003), while good institutions might interact with both physical geography (Frankel et al., 1996; Frankel and Romer, 1999) and the level of political rights for different ethnic groups (Bloom et al., 1998, p. 277–278). Traditionally, institutions are captured by metrics assessing (i) the level of democracy, (ii) macroeconomic stability, (iii) good governance (such as political stability and absence of violence) or (iv) the protection of private property (La Porta et al., 1998).

Apart from food security, other outcomes of the development process include demography (such as population (growth) and age distribution (Bloom et al., 1998)), agglomeration of firms (Lenaerts et al., 2020) and people (urbanisation) (Bloem and De Pee, 2017; Demont, 2013; Headey et al., 2017; Satterthwaite et al., 2010), and economic performance (such as GDP, employment, productivity, etc.) (Dollar et al., 2013). The outcome of interest in this paper is the state of food security and nutrition in the world, which is affected by other development outcomes and the fundamental causes outlined above (FAO et al., 2019). The state of food security and nutrition can be structured as a hierarchy of causes resulting in undernutrition (UNICEF, 1991): (i) basic determinants discussed above (at the country level); (ii) food security consisting of food availability, food access, food utilisation and stability (at the household level); (iii) hunger, such as hidden hunger, chronic hunger and undernourishment (at the individual level); and (iv) undernutrition (or burden of hunger), such as underweight, stunting and wasting (at the individual level) (World Food Programme, 2009).

## 3 Methodology

Based on Clemens (2017), we classify our methodology as a combination of reanalysis (new model specifications) and extension (new data in the form of more years and subsampling)—which are both a type of robustness analysis—of an existing study. GSQ econometrically analysed the drivers of the global burden of chronic and hidden hunger by applying country-level fixed and random effects models on a dataset spread over three points in time. Fixed effects coefficients are typically introduced to control for country-specific characteristics such as culture, history and endowments, which are difficult to measure accurately and change only slowly over time. This reduces bias in the coefficients of the determinants if omitted country-specific characteristics are correlated with the included variables. If the included variables adequately control for country-specific characteristics, the random effects specification is more efficient by correcting for autocorrelation across time, which arises because country-specific characteristics are typically carried over from year to year.

The burden of hunger was measured using Disability-Adjusted Life Years (DALYs) (IHME., 2013a, IHME., 2013b). DALYs can capture the outcome of food insecurity, that is, the burden of disease that is caused by hidden and chronic hunger. More speciﬁcally, hunger-related DALYs capture “person-years lost in a population owing to disability and shortened life” (Stein, 2014) as a consequence of adverse health outcomes associated with the burden of hunger. The burden of hunger (measured in DALYs) can be broken down into cause and risk factors. Cause factors are hunger-related diseases or injuries (that is, nutritional deficiencies) that directly cause death or disability; risk factors (per cause-risk attribution) are hunger-related factors that are causally associated with the probability of a disease or injury.

GSQ included seven determinants as fundamental causes for the global burden of hunger, which can be grouped into four categories: economic performance, demography, institutions and climate (Table 1). GSQ also conducted a Hausman test that showed coefficients to differ significantly between the fixed and random effects specifications. This is neither surprising nor very informative since GSQ did not control for country-level geographies like temperature and market potential. As we have argued in Section 2, geography is a fundamental determinant of economic development and hence food security, which cannot be ignored. Although fixed effects control for country-specific characteristics like geography, they leave no room for interpreting separate effects such as temperature and market potential. Instead, all time-constant characteristics are aggregated at the country-level. A better alternative than merely adding nuisance terms, from which it is difficult to draw any meaningful conclusions, is to attempt to find potential variables that might explain differences in the burden of hunger, including (economic) geography (Beck, 2001).

**Table 1:** Overview of basic determinants of the burden of hunger included by GSQ and this study.

Fundamental causes	GSQ	This study
Economic performance	GDP per capita	GDP per capita
Demography	Share of urban population	Share of urban population
	Share of population aged 0–14 years	
Institutions	Electoral democracy	ICRG risk index
Climate	Average rainfall (mm)	Average rainfall (mm)
	Share of land area in temperate zones	Average temperature (Celsius)
	Share of land area in tropics and subtropics	
Economic geography		Trade openness (exports/GDP)
		Market potential

*Note*: ICRG denotes the International Country Risk Guide.

We propose to expand GSQ's set of four categories with a fifth category: economic geography (Table 1; see Table A3 in the supplementary material for more details). To achieve an optimal balance between (i) capturing all categories to minimize omitted variable bias, and (ii) minimizing multicollinearity among the variables, we drop one of the variables in the demographic category, notably the share of youngsters in the population, and keep the variable “per capita GDP” to avoid omitted variable bias, since we expect all drivers of the burden of hunger to be correlated with per capita GDP. This contained variance inflation factors (VIFs) within the range of 1.2–5.7 with a mean VIF of 2.5, which is largely within the acceptable range (Hair et al., 2011; Sheather, 2009). To capture institutions, we suggest the International Country Risk Guide (ICRG) risk index instead of the binary democracy indicator employed by GSQ for two reasons: (i) the ICRG index integrates economic, financial and political risk (with political dimensions such as macroeconomic stability, ethnic tensions, socioeconomic conditions, democratic accountability and governance); and (ii) a 100-point scale better captures institutional heterogeneity than a binary indicator. To capture climate, we rely solely on rainfall and temperature as these two are the only variables used to construct the Koeppen-Geiger climate classification. Economic geography is finally captured through trade openness (measured as the share of the value of exports in GDP) and market potential (measured as the inverse-distance weighted sum of GDP for all countries). By employing the fifth generation of the GBD dataset (Global Burden of Disease Study 2017) (IHME, 2019), we update and extend the three-point DALY estimates used by GSQ for 1990, 2005 and 2010 (first generation, Global Burden of Disease Study 2010) to a continuous panel with annual data points spanning 1990–2017 for the descriptive analysis, 1990–2015 for the regression analysis on the basic determinants, 1990–2013 for the regression analysis on food supply and 2000–2015 for the regression analysis on health and gender. Different spellings of country names were matched across data sources. Any remaining missing values were dealt with by omitting the respective year-country record. Further details on the data sources used can be found in the supplementary material.

A second drawback of using (group) fixed effects is that it makes slow-changing variables over time (temporally stable or sluggish variables) to appear either statistically or economically insigniﬁcant (Beck, 2001; Beck and Katz, 2001). Unfortunately, many variables of interest related to geography or political economy are almost constant over time. Therefore, “the use of fixed effects will yield odd estimates for coefficients of variables like democracy since the effect of democracy is then “controlled” for [by] the fixed effects” (Beck and Katz, 2001, p. 493). To check whether variables are temporally stable, we calculate their level of time invariance. A simple measure of time invariance can be obtained by calculating the R squared of regressing each variable on the set of country-level dummies. High values are an indicator of multicollinearity between the fixed-effects specification and the variables included. Table 2 shows that the level of time invariance for most variables exceeds 95 per cent. This means that after controlling for country-fixed effects only 5 per cent of variance is left to study the impact of the respective variable on the burden of hunger.

**Table 2:** A measure of time invariance and probit (first-stage) regression estimates predicting countries with a low burden of hunger.

	Time invariance	Probit model
Status of variables listed	Dependent	Independent
*Burden of hunger*		
Log (chronic hunger per capita)	0.986	–1.1727***
		(0.1171)
Log (hidden hunger per capita)	0.982	–4.9844***
		(0.3629)
*Basic determinants*		
Log (GDP per capita, PPP)	0.985	1.1229***
		(0.2460)
Urban population (per cent of total)	0.991	0.0477***
		(0.0096)
ICRG risk index	0.875	0.0875***
		(0.0236)
Average rainfall (mm)	0.955	–0.0020
		(0.0014)
Average temperature (Celsius)	0.998	–0.0565***
		(0.0177)
Trade openness (exports/GDP) (both in US$)	0.859	1.1914***
		(0.4196)
Log (Market potential)	0.776	–0.8611***
		(0.2228)
*Food supply*		
Food supply (kcal/capita/day)	0.960	–0.0019***
		(0.0005)
Cereal production and imports (kg/capita/year)	0.932	0.0006***
		(0.0002)
Supply of vegetables and fruits (kcal/capita/day)	0.934	0.0006
		(0.0019)
Supply of roots and tubers (kcal/capita/day)	0.989	0.0039**
		(0.0017)
*Health and gender*		
Ratio of female to male life expectancy at birth	0.970	–9.1275***
		(2.4324)
Access to safe water (per cent of population)	0.982	–0.2687***
		(0.0333)
Number of observations	1734	1734
Number of countries	195	195

*Notes*: GDP, gross domestic product. PPP, purchasing power parity. ***, ** and * denote signiﬁcance at the 1, 5 and 10 per cent level, respectively. Time invariance is calculated as the R squared of a pooled OLS with country-level dummies. For the probit regression, all years between 2000 and 2013 are included.

Another potential problem is the fact that many high-income countries show DALY estimates that are near-zero and therefore are difficult to compare with middle- and low-income countries in terms of the burden of hunger. The burden of hunger in high-income countries is—albeit not zero—marginal compared to that in low-income countries. In addition, by being often located in the Western world, high-income countries differ markedly from middle- and low-income countries. We can formally test this using a binomial regression on a dummy indicating whether a country suffers substantially from hunger (Blum and Goldfarb, 2006). Both hidden and chronic hunger show a heavily right-skewed distribution with a density peak near zero (see Figure A2 in the supplementary material). We use these density peaks as cutoff values to define the set of hunger-afflicted countries: a country is assumed to be free from hunger if its burden of hunger has been below the density peak for both hidden and chronic hunger for every year included in the dataset (see Table A2 in the supplementary material for a list of countries). Using a probit model where the dependent variable is equal to one if the country is not afflicted by hunger, Table 2 indicates that hunger-afflicted countries differ from the rest of the world in terms of per capita GDP, urbanisation, ICRG risk index, average temperature, economic geography, the supply of different types of food, gender equality and public health.

Given these concerns, we suggest (pooled) OLS estimates with clustered (or panel correct) standard errors (Beck, 2001; Beck and Katz, 2001). We allow for a small modification by adding yearly fixed effects to control for macroeconomic shocks (such as the 2007–2008 peak in food prices) and calculating two-way clustered standard errors. In addition, we calculate coefficient estimates conditional on countries suffering substantially from hunger (that is, excluding the subsample of hunger-free countries defined above). These conditional effects provide more targeted and relevant insights for policymakers working in middle- and low-income countries.

## 4 Results

### 4.1 Descriptive evidence

**Fig. 1:**
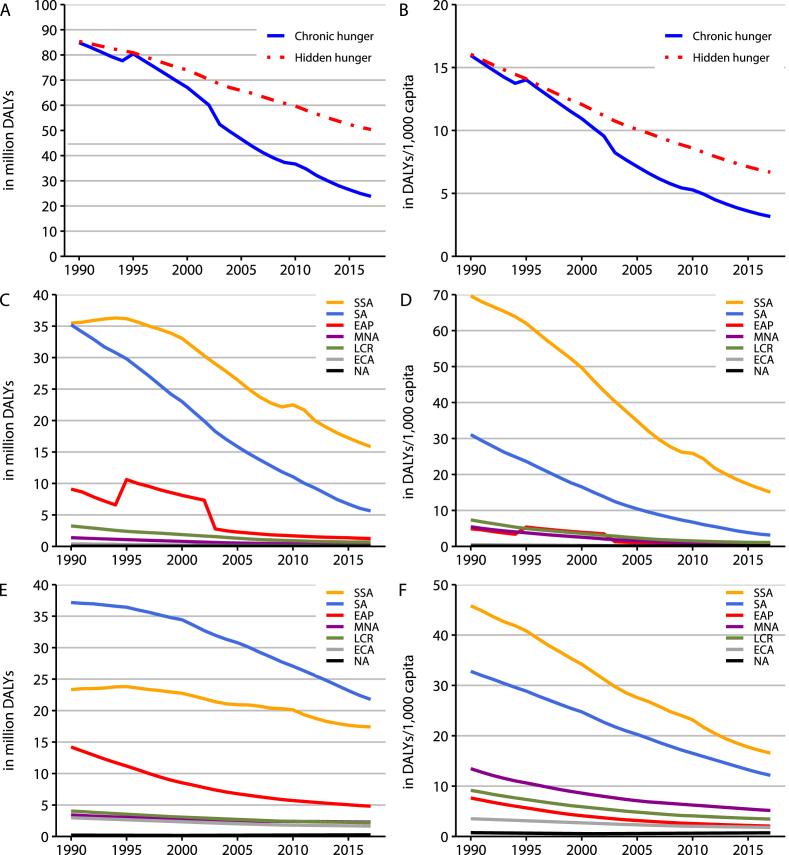
Development of the burden of hunger between 1990 and 2017 by region. (A) DALYs (million) lost due to chronic and hidden hunger globally. (B) DALYs per 1000 capita lost due to chronic and hidden hunger globally. (C) DALYs (million) lost due to chronic hunger regionally. (D) DALYs per 1000 capita lost due to chronic hunger regionally. (E) DALYs (million) lost due to hidden hunger regionally. (F) DALYs per 1000 capita lost due to hidden hunger regionally. *Notes*: Per capita values represent ratios of global or regional DALYs over population, multiplied by a factor of 1000. SSA, Sub-Saharan Africa. SA, South Asia. EAP, East Asia and Paciﬁc. MNA, Middle East and North Africa. LCR, Latin America and the Caribbean. ECA, Europe and Central Asia. NA, North America. The spike in the EAP curve in C and D is caused by the 1994–1998 North Korean famine. Source: IHME (2019).

Between 1990 and 2017, the total burden of chronic hunger reduced by 72 per cent, while the total burden of hidden hunger declined by 41 per cent (Fig. 1). Expressed per capita, the relative burden of chronic hunger dropped by 80 per cent, while the relative burden of hidden hunger shrunk by 58 per cent. In contrast with GSQ, we find DALY estimates that are 25–50% smaller, particularly because we find chronic hunger to be less prevalent than hidden hunger across the entire timeframe opposite to GSQ. This difference is likely the result of substantial improvements in the estimation of cause and risk factors. For example, while unspeciﬁed anaemia was mapped to iron-deﬁciency anaemia in the Global Burden of Disease Study 2010, this was no longer the case for later generations of the GBD dataset (GBD 2013 collaborators, 2015). In line with GSQ, we find that the burden of chronic hunger has fallen more rapidly than the burden of hidden hunger.

**Fig. 2:**
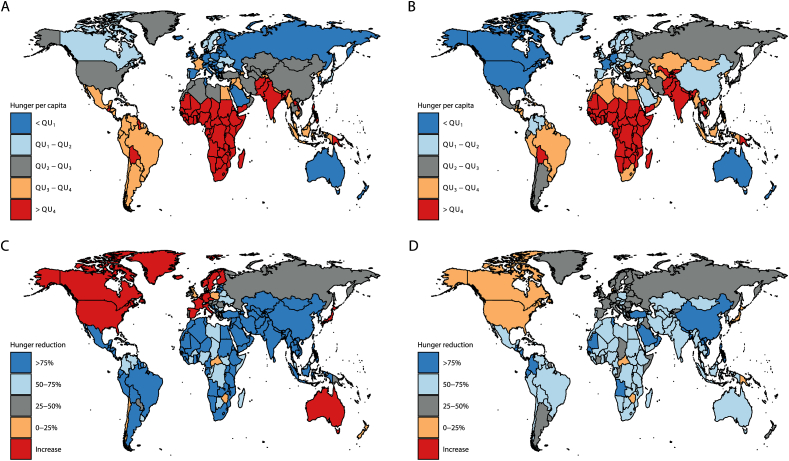
Map of the relative burden of hunger expressed in quintiles (QU). (A) Chronic hunger per capita in 2017. (B) Hidden hunger per capita in 2017. (C) Relative change in chronic hunger per capita between 1990 and 2017. (D) Relative change in hidden hunger per capita between 1990 and 2017. Source: IHME (2019).

Comparing across regions, we find that Sub-Saharan Africa is the region suffering the most from the burden of chronic hunger, while South Asia features the highest burden of hidden hunger. Expressed per capita, Sub-Saharan Africa clearly suffers more than any other region from both burdens. A similar picture emerges from Fig. 2, showing a map of 2017 values and changes between 1990 and 2017 in the relative burden of hunger. In 2017, Sub-Saharan Africa and South Asia still suffered substantially more from the relative burden of hunger than the rest of the world (upper quintiles). Other countries in this group include Bolivia, Yemen, Laos and Papua New Guinea.

The largest contributions to the reduction in the global burden of chronic hunger between 1990 and 2017 can be attributed to India (33 per cent), Bangladesh (10 per cent), Ethiopia (6 per cent), China (6 per cent) and Nigeria (5 per cent), while in the case of hidden hunger, the largest contributors to its global alleviation are India (29 per cent), China (14 per cent), Bangladesh (7 per cent), Pakistan (6 per cent) and Indonesia (5 per cent). Although Sub-Saharan Africa and South Asia still suffered the most from the relative burden of hunger in 2017, most of these countries experienced significant reductions in the relative burden of chronic (>75 per cent) and hidden hunger (>50 per cent). However, not every country reported a reduction in the burden of hunger as some high-income countries experienced an increase in the relative burden of chronic hunger. This additionally indicates that care should be taken when assessing the determinants of the burden of hunger for the entire sample of countries worldwide.

**Fig. 3:**
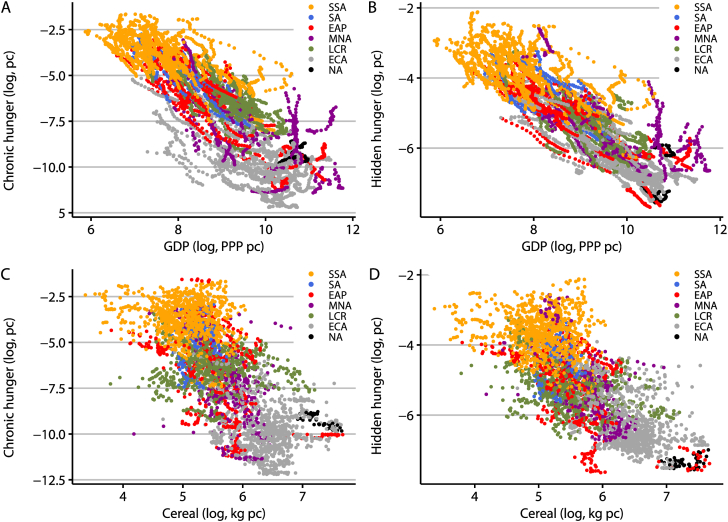
Scatter plot of the burden of hunger (in log, per capita) versus economic performance and cereal availability (sum of imports and production; both in log, per capita) by region. (A) Chronic hunger versus GDP. (B) Hidden hunger versus GDP. (C) Chronic hunger versus the sum of imported and produced cereal. (D) Hidden hunger versus the sum of imported and produced cereal. *Notes*: SSA, Sub-Saharan Africa. SA, South Asia. EAP, East Asia and Paciﬁc. MNA, Middle East and North Africa. LCR, Latin America and the Caribbean. ECA, Europe and Central Asia. NA, North America. All years between 1990 and 2017 are included. Source: FAOSTAT (2018), IHME (2019), UN DESA (2019) and World Bank (2019).

Before looking at the regression results, we focus on two factors that affect the burden of hunger and are at the forefront of development policy: per capita GDP and per capita cereal availability (sum of imports and production). The burden of chronic and hidden hunger displays a strongly downward-sloping trend for both factors (Fig. 3). Sub-Saharan Africa features the lowest levels of per capita GDP and per capita cereal availability and correspondingly the highest burden of hunger levels. Countries in East Asia, the Paciﬁc, the Middle East and North Africa exhibit the largest heterogeneity of per capita GDP and cereal availability.

### 4.2 Determinants of the global burden of hunger

Table 3 lists the effect of different basic determinants on the burden of hunger for (i) the full sample of countries (columns 1, 3, 1a and 3a) and the sample of countries suffering substantially from hunger (columns 2, 4, 2a and 4a) and (ii) with (columns 3, 4, 3a and 4a) and without (columns 1, 2, 1a and 2a) controlling for per capita GDP. The results of the regression analysis suggest that per capita GDP (gross domestic product) is still a strong driver of the burden of hunger: a one per cent increase in GDP is associated with a one per cent decrease in the burden of chronic hunger and a 0.6 per cent decrease in the burden of hidden hunger (Table 3). These effects are approximately twice the ones reported by GSQ, indicating that the role of economic growth in alleviating hunger remained important after 2010 (Dollar et al., 2013).

**Table 3:** Associations of basic determinants with the burden of chronic and hidden hunger.

	Burden of chronic hunger					Burden of hidden hunger			
	(1)	(2)	(3)	(4)		(1a)	(2a)	(3a)	(4a)
Log (GDP per capita, PPP)			–1.143***	–0.995***				–0.615***	–0.581***
			(0.176)	(0.163)				(0.071)	(0.073)
Urban population (per cent of total)	–0.042***	–0.040***	–0.005	–0.007		–0.022***	–0.020***	–0.003	–0.002
	(0.006)	(0.006)	(0.008)	(0.007)		(0.003)	(0.003)	(0.003)	(0.004)
ICRG risk index	–0.060***	–0.047***	–0.016	–0.012		–0.030***	–0.025***	–0.006	–0.002
	(0.011)	(0.010)	(0.011)	(0.009)		(0.006)	(0.006)	(0.005)	(0.005)
Average rainfall (mm)	0.000	–0.000	–0.000	–0.001		–0.002***	–0.002***	–0.002***	–0.003***
	(0.001)	(0.001)	(0.001)	(0.001)		(0.001)	(0.001)	(0.001)	(0.001)
Average temperature (Celsius)	0.077***	0.069***	0.083***	0.081***		0.042***	0.037***	0.043***	0.041***
	(0.017)	(0.014)	(0.016)	(0.013)		(0.008)	(0.009)	(0.007)	(0.008)
Trade openness (exports/GDP) (both in US$)	–0.386	–0.770	0.008	–0.341		0.088	–0.088	0.364**	0.238
	(0.452)	(0.479)	(0.398)	(0.370)		(0.226)	(0.251)	(0.180)	(0.180)
Log (Market potential)	–1.223***	–1.857***	–0.855***	–1.481***		–0.397***	–0.585***	–0.214	–0.387***
	(0.338)	(0.312)	(0.303)	(0.289)		(0.148)	(0.146)	(0.139)	(0.141)
Full (F) or subsample (S)	F	S	F	S		F	S	F	S
Number of observations	3280	2726	3203	2655		3280	2726	3203	2655
Number of countries	135	112	132	109		135	112	132	109
R-squared	0.76	0.77	0.81	0.82		0.76	0.71	0.83	0.79

*Notes*: These are results from time-fixed effects panel regressions with annual data from 1990 until 2015 on the subsample of countries listed in Table A2. Two-way clustered standard errors are shown in parentheses. The dependent variable is the logarithm of DALYs per capita lost due to chronic hunger (models 1–2) and hidden hunger (models 1a–2a). GDP, gross domestic product. PPP, purchasing power parity. ***, ** and * denote signiﬁcance at the 1, 5 and 10 per cent level, respectively.

Further, our results show that urbanisation and good governance have a negative but statistically insignificant effect on the burden of hunger once per capita GDP is controlled for, which is in contrast with the significantly negative effect found by GSQ. A possible explanation for this might be that although urbanisation and governance positively affect food security, they are only indirect effects (Bezemer and Headey, 2008; Ross, 2006). The reason why increasing urbanisation and governance appears to lower the burden of hunger significantly in GSQ is that they control for each country's local situation through the use of country-fixed effects. However, on a global scale, governance and urbanisation are only correlated with economic development and therefore, not directly driving eradication of hunger.

In terms of environmental factors, higher levels of rainfall have a statistically significantly negative effect on the burden of hidden hunger while higher temperature levels have a statistically significantly positive effect on both the burden of chronic and hidden hunger. As pointed out by GSQ, this is a troubling finding given that many middle- and low-income countries already face high temperatures and water stress, and climate change is likely to negatively affect many regions in Africa (Niang et al., 2014) and Asia (Hijioka et al., 2014). Although these results are intuitively understood as determinants of the production environment, care should be taken when inferring causality since climate variables like rainfall and temperature are correlated with geography and therefore, might pick up hidden variables. For example, precipitation and temperature peak around the equator but so does ethnolinguistic fragmentation as well (World Bank, 2008, p. 104).

To capture economic geography, we included a country's physical centrality to markets (market potential) and openness to trade. Trade openness only appears a statistically significant driver for the burden of hidden hunger for the full sample of countries and when controlling for GDP (column 3a). However, the positive effect is likely a spurious finding (Brooks and Matthews, 2015) as trade openness is no longer statistically significant in the subsample specification (column 4a). Higher levels of market potential are associated with a lower burden of chronic and hidden hunger, although this effect becomes insignificant when we control for GDP and consider the full sample. This implies that a country's standard of living as well as that of its neighbouring countries affect the burden of hunger. Regional clusters in per capita GDP (see Figure A3 in the supplementary material) overlap with the regional trends in the burden of hunger shown in Fig. 2. Therefore, spatial spillovers in prosperity are one possible explanation for the existence of regional clusters in the burden of hunger. Table 3 indicates that the full sample specification (unconditional effects) differs mainly from the subsample specification (conditional effects) regarding the geographic variables trade openness and market potential. Since we focus on countries facing high burdens of hunger, we are interested in the conditional effects; therefore, we continue with the subsample specification for the remainder of the analysis.

To isolate the effect of the data, we replicated the exact model used by GSQ but with the newer data (see uneven columns in Table A4 in the supplementary material). In comparison with the original results by GSQ, most effects are comparable in size and significance, which points to the improved methodology as the main reason for the differences in regression results in Table 3 with GSQ. Exceptions are the climate variables since the newer data no longer yields a significant effect of average rainfall and only significant effects of climate zones for the burden of chronic hunger (in GSQ, only land area in temperate zones yields significant effects, for both chronic and hidden hunger). The temperate zone effect from GSQ in the estimates of the random effects seems to be captured by average temperature (even columns in Table A4), that is, higher temperatures (thus, less temperate zones) are associated with higher burdens of hunger. The effect is reversed in the fixed effects estimates, which can be explained by the low time variance of climate variables such as rainfall and temperature.

### 4.3 The role of food supply

Table 4 presents evidence on the role of different types of food supply (food availability) on the burden of hunger. Following GSQ, we find that a higher total food supply (kcal/capita/day), in terms of calories from both plant and animal products in general, is associated with a lower burden of chronic and hidden hunger, even after controlling for per capita GDP. Our regression results indicate that all types of animal products, except fish, are negatively associated with the burden of chronic and hidden hunger. In terms of plant products, cereals and pulses matter for the burden of chronic but not hidden hunger. The supply of vegetables and fruits is significantly associated with the burden of chronic and hidden hunger, while there is no statistically significant effect for roots and tubers. These findings for plant products are in line with general views from the field of food science but contrast with the results reported by GSQ (that is, controlling for per capita GDP, they find no effect for cereals and vegetables and fruits, a statistically significantly negative effect for pulses on the burden of hidden hunger and a statistically significantly negative effect for roots and tubers on the burden of chronic and hidden hunger). Our results for cereals are not surprising as cereals are the most important source of calories for the poor but feature low nutritional density in terms of micro-nutrients. Roots and tubers do not have a statistically significant impact on chronic and hidden hunger (when controlled for GDP), perhaps because they mainly provide dietary energy (Sharma et al., 2016). Vegetables and fruits are generally rich in proteins and nutrients explaining their significant impact on chronic and hidden hunger (Sanchez, 2020), although some vegetables such as pulses are rich in proteins rather than micronutrients.

**Table 4:** Associations of the food supply with the burden of chronic and hidden hunger.

	Burden of chronic hunger			Burden of hidden hunger	
Models with total per capita food supply	(1)	(2)		(1a)	(2a)
Log (GDP per capita, PPP)		–1.0104***			–0.5592***
		(0.1195)			(0.0593)
Food supply (kcal/capita/day)	–0.0039***	–0.0021***		–0.0017***	–0.0007***
	(0.0002)	(0.0003)		(0.0001)	(0.0001)
Number of observations	3415	3276		3415	3276
Number of countries	145	143		145	143
R–squared	0.55	0.67		0.51	0.70

Disaggregation of food supply (A)	(3)	(4)		(3a)	(4a)
Log (GDP per capita, PPP)		–0.6858***			–0.4193***
		(0.1318)			(0.0624)
Supply of animal products (kcal/capita/day)	–0.0062***	–0.0042***		–0.0028***	–0.0016***
	(0.0004)	(0.0006)		(0.0001)	(0.0002)
Supply of plant products (kcal/capita/day)	–0.0022***	–0.0015***		–0.0008***	–0.0004***
	(0.0003)	(0.0003)		(0.0001)	(0.0001)
Number of observations	3415	3276		3415	3276
Number of countries	145	143		145	143
R–squared	0.66	0.71		0.65	0.74

Disaggregation of food supply (B)	(5)	(6)		(5a)	(6a)
Log (GDP per capita, PPP)		–0.5600***			–0.3172***
		(0.1172)			(0.0564)
Supply of meat (kcal/capita/day)	–0.0030***	–0.0015		–0.0020***	–0.0012***
	(0.0010)	(0.0010)		(0.0004)	(0.0004)
Supply of ﬁsh (kcal/capita/day)	–0.0016	0.0002		–0.0009	0.0001
	(0.0023)	(0.0021)		(0.0010)	(0.0009)
Supply of eggs (kcal/capita/day)	–0.0541***	–0.0406***		–0.0279***	–0.0209***
	(0.0068)	(0.0067)		(0.0026)	(0.0026)
Supply of milk (kcal/capita/day)	–0.0061***	–0.0055***		–0.0013***	–0.0010**
	(0.0013)	(0.0012)		(0.0005)	(0.0004)
Supply of cereals (kcal/capita/day)	–0.0007**	–0.0007**		–0.0001	–0.0001
	(0.0003)	(0.0003)		(0.0001)	(0.0001)
Supply of pulses (kcal/capita/day)	0.0059***	0.0065***		0.0011	0.0010
	(0.0020)	(0.0018)		(0.0009)	(0.0009)
Supply of vegetables and fruits (kcal/capita/day)	–0.0044***	–0.0028***		–0.0015***	–0.0007*
	(0.0009)	(0.0009)		(0.0004)	(0.0004)
Supply of roots and tubers (kcal/capita/day)	0.0005	0.0000		0.0007***	0.0004
	(0.0005)	(0.0005)		(0.0002)	(0.0002)
Number of observations	3393	3254		3393	3254
Number of countries	144	142		144	142
R–squared	0.72	0.76		0.74	0.78

*Notes*: These are results from time-fixed effects panel regressions with annual data from 1990 until 2013 on the subsample of countries listed in Table A2. Two-way clustered standard errors are shown in parentheses. The dependent variable is the logarithm of DALYs per capita lost due to chronic hunger (models 1–6) and hidden hunger (models 1a–6a). GDP, gross domestic product. PPP, purchasing power parity. ***, ** and * denote signiﬁcance at the 1, 5 and 10 per cent level, respectively.

Table 5 provides further evidence by indicating that both higher levels of cereal production and imports, which are a proxy for food availability, help to lower the burden of chronic and hidden hunger. The effect on hidden hunger is smaller (in line with cereals' nutritional composition) but still significant. The latter might be due to the imperfectness of cereal production and imports as proxies for cereal availability, that is, production and import of cereals might be correlated with the production and imports of micronutrient-rich foods. The contribution of cereal imports to reducing the global burden of chronic hunger is almost three times higher than the contribution of domestic cereal production. This highlights the potential role of trade (imports) to supplement the inadequate production of food and hence, strengthen global food security (Saverimuttu and Rempel, 2004). At the same time, however, reliance on food imports makes net food importers vulnerable to market fluctuations such as the 2007–2008 global food crisis (Moseley et al., 2010; Seck et al., 2010). After controlling for per capita GDP, both contributions decline and converge. This suggests that it is not trade openness per se but instead purchasing power that affects the burden of hunger, which confirms our previous findings.

**Table 5:** Associations of cereals production and imports with the burden of chronic and hidden hunger.

	Burden of chronic hunger			Burden of hidden hunger	
	(1)	(2)		(1a)	(2a)
Log (GDP per capita, PPP)		–1.2171***			–0.6426***
		(0.1092)			(0.0461)
Cereals production (kg/capita/year)	–0.0054***	–0.0032***		–0.0025***	–0.0013***
	(0.0006)	(0.0005)		(0.0003)	(0.0002)
Cereals import quantity (kg/capita/year)	–0.0131***	–0.0053***		–0.0056***	–0.0014***
	(0.0018)	(0.0015)		(0.0007)	(0.0005)
Number of observations	3343	3204		3343	3204
Number of countries	142	140		142	140
R-squared	0.43	0.66		0.42	0.72

*Notes*: These are results from time-fixed effects panel regressions with annual data from 1990 until 2013 on the subsample of countries listed in Table A2. Two-way clustered standard errors are shown in parentheses. The dependent variable is the logarithm of DALYs per capita lost due to chronic hunger. GDP, gross domestic product. PPP, purchasing power parity. ***, ** and * denote significance at the 1, 5 and 10 per cent level, respectively.

### 4.4 The role of health and gender

Table 6 presents evidence on the role of gender equality and public health (which might affect food access and food utilisation) on the burden of hunger. We find that female school enrolment and the female-to-male life expectancy ratio are both associated with a signiﬁcantly lower burden of hidden hunger. At the same time, access to safe water and improved sanitation and immunisation have a statistically significantly negative effect on the burden of chronic and hidden hunger. In comparison to GSQ, our findings emphasise the importance of public health while only correlating gender equality with the burden of hidden hunger. Given the ongoing Covid-19 pandemic, improving the understanding of the role of public health and gender equality on hunger is a priority towards assessing the impact of the pandemic on middle- and low-income countries. Our results are consistent with the HLPE (2017) food systems framework for diets and nutrition, which explicitly frames women empowerment as a direct driver of consumer behaviour and diets and indirectly through the food environment. The effect of income (GDP per capita) on diets is conditioned by the food environment, which determines the availability, access, affordability, promotion, advertising, information, quality and safety of food (HLPE, 2017). Increased income can reduce hidden hunger where the food environment enables its use on healthy diets, and women's empowerment and gender equality are important mediators of this impact pathway (Herforth and Ahmed, 2015).

**Table 6:** Associations of gender equality and public health with the burden of chronic and hidden hunger.

	Burden of chronic hunger					Burden of hidden hunger			
	(1)	(2)	(3)	(4)		(1a)	(2a)	(3a)	(4a)
Log (GDP per capita, PPP)		–0.5595***		–0.4772***			–0.3629***		–0.2879***
		(0.1588)		(0.1295)			(0.0568)		(0.0501)
School enrollment, primary female (per cent net)	–0.0039	0.0031				–0.0109***	–0.0117***		
	(0.0076)	(0.0063)				(0.0030)	(0.0027)		
Ratio of female to male life expectancy at birth	–0.6397	–3.2965				–2.8259**	–4.0452***		
	(3.5825)	(3.2254)				(1.2854)	(1.3484)		
Access to safe water (per cent of population)	–0.0206**	–0.0134	–0.0236***	–0.0177***		–0.0149***	–0.0086**	–0.0135***	–0.0092***
	(0.0093)	(0.0087)	(0.0064)	(0.0065)		(0.0047)	(0.0037)	(0.0032)	(0.0034)
Access to improved sanitation (per cent of population)	–0.0484***	–0.0355***	–0.0468***	–0.0356***		–0.0158***	–0.0075***	–0.0190***	–0.0125***
	(0.0059)	(0.0061)	(0.0038)	(0.0046)		(0.0026)	(0.0023)	(0.0019)	(0.0021)
Immunisation DPT (per cent of children ages 12–23 months)	–0.0165***	–0.0191***	–0.0137***	–0.0148***		0.0005	–0.0001	–0.0058***	–0.0074***
	(0.0057)	(0.0063)	(0.0042)	(0.0047)		(0.0029)	(0.0029)	(0.0020)	(0.0023)
Number of observations	1454	1415	2527	2442		1454	1415	2527	2442
Number of countries	147	144	162	158		147	144	162	158
R-squared	0.73	0.75	0.76	0.78		0.78	0.83	0.77	0.80

*Notes*: These are results from time-fixed effects panel regressions with annual data from 2000 until 2015 on the subsample of countries listed in Table A2. Two-way clustered standard errors are shown in parentheses. The dependent variable is the logarithm of DALYs per capita lost due to chronic hunger (models 1–4) and hidden hunger (models 1a–4a). GDP, gross domestic product. PPP, purchasing power parity. DPT, diphtheria, pertussis, tetanus. ***, ** and * denote signiﬁcance at the 1, 5 and 10 per cent level, respectively.

## 5 Conclusion

Gödecke et al. (2018, 2019) (GSQ) generated important insights into the trends and determinants of the global burden of chronic and hidden hunger based on a dataset spread over three discrete points in time: 1990, 2005 and 2010. We reanalysed the GSQ study using a more robust specification and extended the data to a panel of countries suffering substantially from hunger, continuously spanning the period 1990–2017. We demonstrate that after 2010, significant reductions in the burden of chronic and hidden hunger were achieved. Since 1990, the relative burdens of chronic and hidden hunger have decreased by 80 and 58 per cent, respectively. Countries typically associated with hunger such as India, Bangladesh and Ethiopia are also among those contributing most to combatting hunger globally. Over the last three decades, global and regional trends show that the burden of hidden hunger has always exceeded the burden of chronic hunger, which contrasts GSQ's earlier findings. In line with GSQ, we find that the burden of chronic hunger is falling more rapidly than the burden of hidden hunger, which suggests that policies should increasingly target hidden hunger.

Following GSQ, we also assessed the impact of country-level determinants on the burden of chronic and hidden hunger. We provide evidence on the importance of spatial spillovers in GDP, which is in line with the existence of supranational clusters in the burden of hunger. Because most countries do not suffer from hunger in isolation, reducing hunger in one country without intervening in neighbouring countries will not suffice to eradicate hunger in the long-run. Development programs with a strong regional focus might be needed. We confirm the effect of per capita GDP and climate in line with GSQ. We find negative but statistically insignificant effects of urbanisation and governance; this might indicate that although both factors can contribute towards lowering the burden of hunger (in line with the literature), on a global scale, they are indirect effects.

Contrasting GSQ, we find that after controlling for per capita GDP, the import and production of cereals and the supply of vegetables (including pulses) and fruits matter in the alleviation of the burden of chronic and hidden hunger; we find no statistically significant impact of root and tubers. These updated conclusions are important as previous findings were not in line with nutritional compositions of the respective food supplies and had far-reaching implications for development policy, especially for cereals. In terms of public health, our results largely follow GSQ, although ours indicate that gender equality only affects hidden hunger and not chronic hunger. Given the ongoing Covid-19 pandemic, improving the understanding of the role of public health and gender equality on hunger is a priority towards assessing the impact of the pandemic on middle- and low-income countries.

Most determinants have larger eﬀects on the burden of chronic hunger than on the burden of hidden hunger, which corroborates the steeper decline in the burden of chronic hunger. As a result, hidden hunger has surpassed chronic hunger over the last three decades. This means that policy makers should prioritize (i) enhancement of micronutrient density of cereals through biofortification and industrial fortification; (ii) diversification of production systems and consumer diets; and (iii) development of nutrition-sensitive food value chains, in which the food environment, women's empowerment and gender equality play crucial mediating roles.

## Declaration of competing interest

The authors declare that they have no known competing financial interests or personal relationships that could have appeared to influence the work reported in this paper.
