# Supplementary material for: The global burden of chronic and hidden hunger revisited: New panel data evidence spanning 1990–2017
Source: Glob Food Sec. 2021 Mar;28:100480. doi: 10.1016/j.gfs.2020.100480 (PMC7937785; doi:10.1016/j.gfs.2020.100480)
Supplement: Supplementary material [file mmc1.pdf]

SUPPLEMENTARY MATERIAL

The global burden of chronic and hidden hunger revisited: New panel data evidence spanning 1990–2017

Bert Lenaerts[1*] and Matty Demont[2]

[1] UHasselt, Centre for Environmental Sciences, Hasselt, Belgium.

[2] International Rice Research Institute (IRRI), Metro Manila, Philippines.

[*] Corresponding author <bert.lenaerts@uhasselt.be>

Overview

Introduction

Text A1. Calculations of the Burden of Chronic and Hidden Hunger

Text A2. Statistical software

Table A1. List of different risk and cause factors related to the burden of hunger.

Figure A1. Development of the burden of hunger between 1990 and 2017 by risk or cause factor.

Figure A2. Kernel density plot of the per capita burden of chronic and hidden hunger.

Figure A3. Map of per capita GDP expressed in quintiles (QU) in 2017.

Table A2. List of countries.

Table A3. Definitions of variables and data sources that differ from GSQ.

References

Introduction

This supplementary appendix provides material not reported in the main paper for brevity. This appendix is not designed to stand alone or to replace the reading of the main paper. For a full understanding, it is recommended to read first Gödecke, Stein and Qaim (2018) (hereafter referred to as GSQ) and the corrected accompanying supplementary material in Gödecke et al. (2019).

Text A1. Calculations of the Burden of Chronic and Hidden Hunger

Because in the GBD 2010 (IHME, 2013a, 2013b) not all age groups were available, GSQ used a combination of risk and cause factors (Equations 1-2). Using the GBD 2017 (IHME, 2019), it is possible to calculate the burden of hunger for risk (Equations 3-4) or cause factors (Equations 5-6) only. Since results differed only marginally[1], we only presented results based on Equations 1-2 (following GSQ).

(1) $CH_A = (PEM_{c,A} - PEM_{c,U5}) + Underweight_{r,U5}$

(2) $HH_A = IDD_{c,A} + FeD_{r,A} + (VAD_{c,A} - VAD_{c,U5}) + VAD_{r,U5} + ZnD_{r,U5} + OtherDef_{c,A}$

[1] Results available from the authors upon request.

(3) $CH_A = Underweight_{r,A}$

(4) $HH_A = IDD_{c,A} + FeD_{r,A} + VAD_{r,A} + ZnD_{r,A} + OtherDef_{c,A}$

(5) $CH_A = PEM_{c,A}$

(6) $HH_A = IDD_{c,A} + FeD_{c,A} + VAD_{c,A} + ZnD_{r,A} + OtherDef_{c,A}$

Table A1. List of different risk and cause factors related to the burden of hunger.

$Underweight_{r,U5}$	disease burden (measured in DALYs lost) attributable to the risk factor childhood underweight for children under 5
$ZnD_{r,U5}$	disease burden (measured in DALYs lost) attributable to the risk factor zinc deficiency for children under 5
$VAD_{r,U5}$	disease burden (measured in DALYs lost) attributable to the risk factor vitamin A deficiency—including the burden of vitamin A deficiency and also intestinal infectious diseases and measles—for children under 5
$FeD_{r,A}$	disease burden (measured in DALYs lost) attributable to the risk factor iron deficiency—including the burden of iron-deficiency anaemia—for persons of all ages
$PEM_{c,A}$	DALYs lost due to protein-energy malnutrition for persons of all ages
$PEM_{c,U5}$	DALYs lost due to protein-energy malnutrition for children under 5
$IDD_{c,A}$	DALYs lost due to iodine deficiency for persons of all ages
$OtherDef_{c,A}$	DALYs lost due to other nutritional deficiencies for persons of all ages
$VAD_{c,A}$	DALYs lost due to vitamin A deficiency for persons of all ages
$VAD_{c,U5}$	DALYs lost due to vitamin A deficiency for children under 5
CH_A	burden of chronic hunger for all age groups
HH_A	burden of hidden hunger for all age groups

Text A2. Statistical software

The analysis was conducted in R (R Core Team, 2020): data cleaning was performed using different *tidyverse* packages (v 1.2.1; Wickham, 2020), figures were produced using the package *ggplot2* (v 1.2.1; Wickham, 2020), regression analyses were conducted using the package *plm* (v 2.2-0; Croissant et al., 2019) and regression tables were produced using the package *texreg* (v 1.36.23; Leifeld, 2017, 2013).

Supplementary Figures and Tables

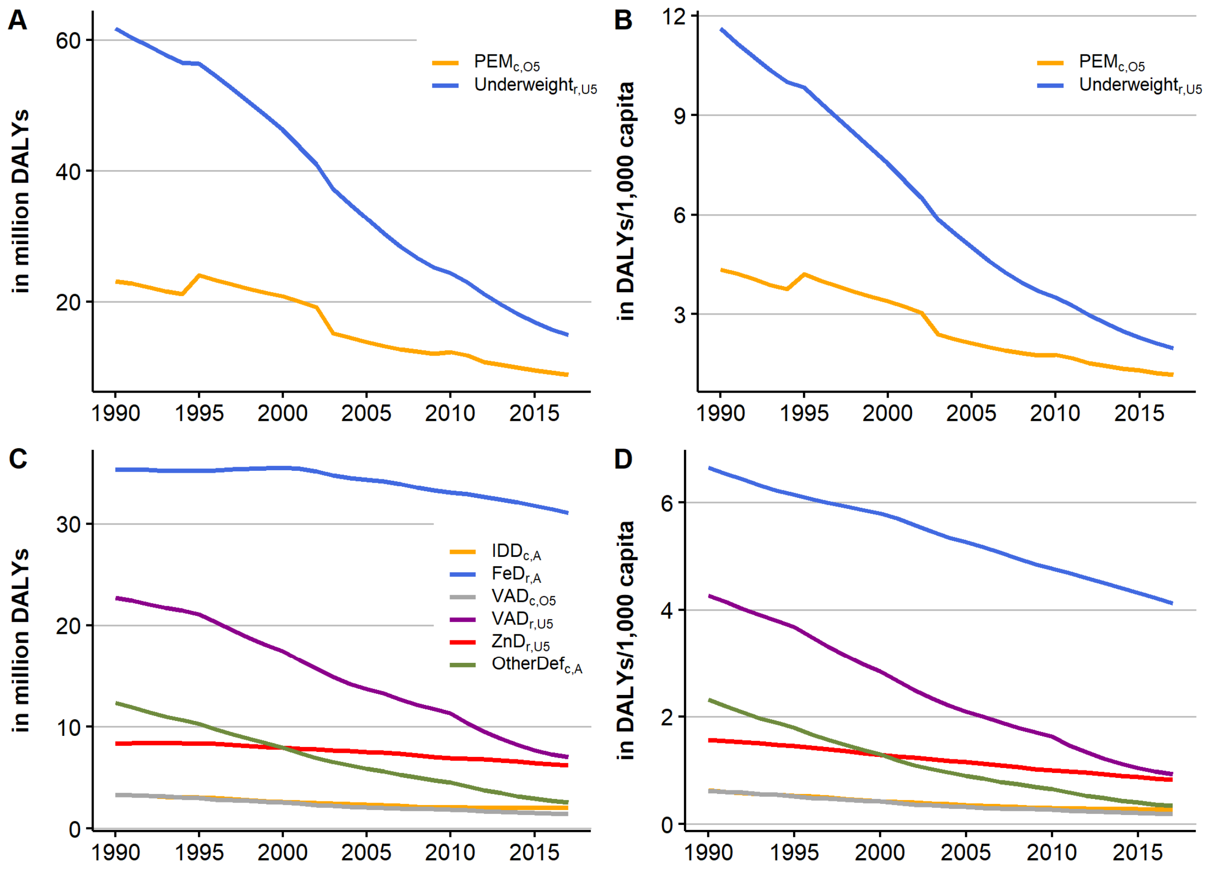

Figure A1. Development of the burden of hunger between 1990 and 2017 by risk or cause factor. (A) DALYs (million) lost due to chronic hunger. (B) DALYs per 1,000 capita lost due to chronic hunger. (C) DALYs (million) lost due to hidden hunger. (D) DALYs per 1,000 capita lost due to hidden hunger.

Notes: DALYs, Disability-Adjusted Life Years. PEM, protein-energy malnutrition. IDD, iodine deficiency. FeD, iron deficiency. VAD, vitamin A deficiency. ZnD, zinc deficiency. OtherDef, other nutritional deficiencies. The subscript A refers to all ages, U5 to children under five and O5 to persons aged five years and older.

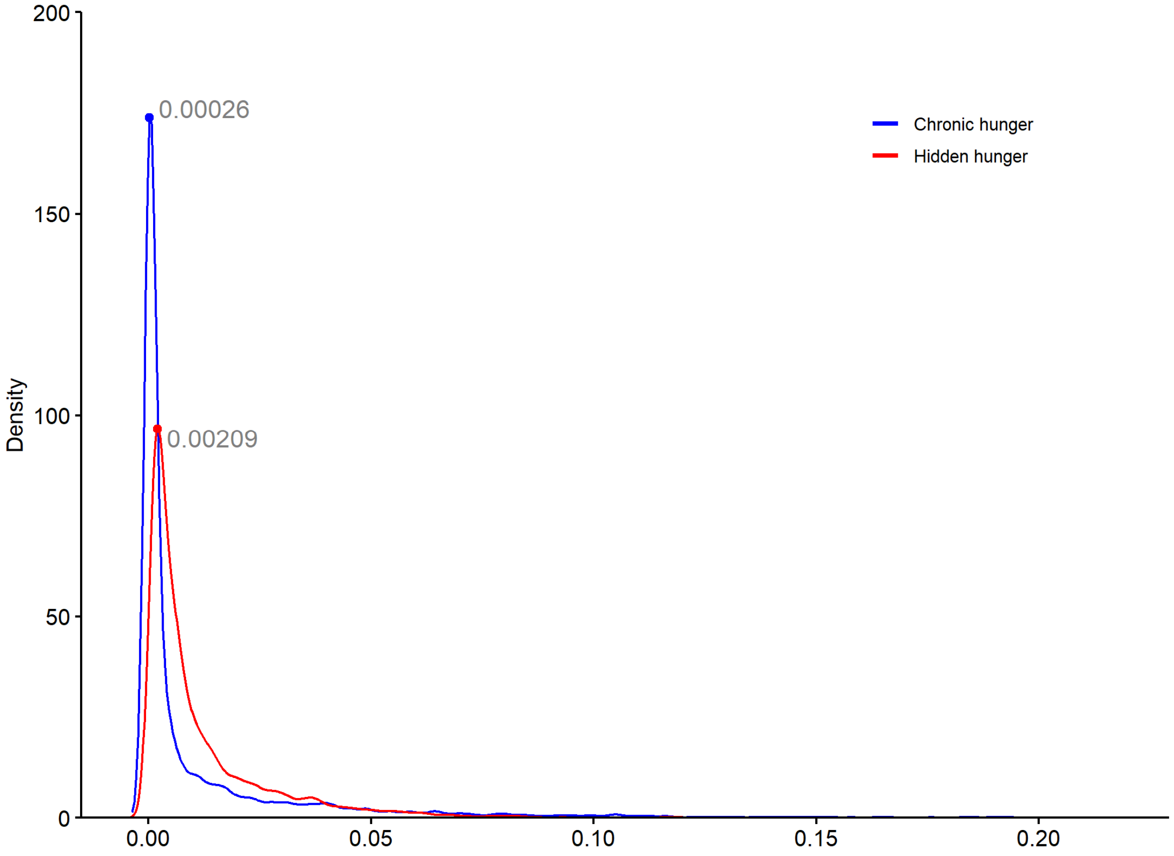

Figure A2. Kernel density plot of the per capita burden of chronic and hidden hunger.
Notes: Dots indicate the density peak.

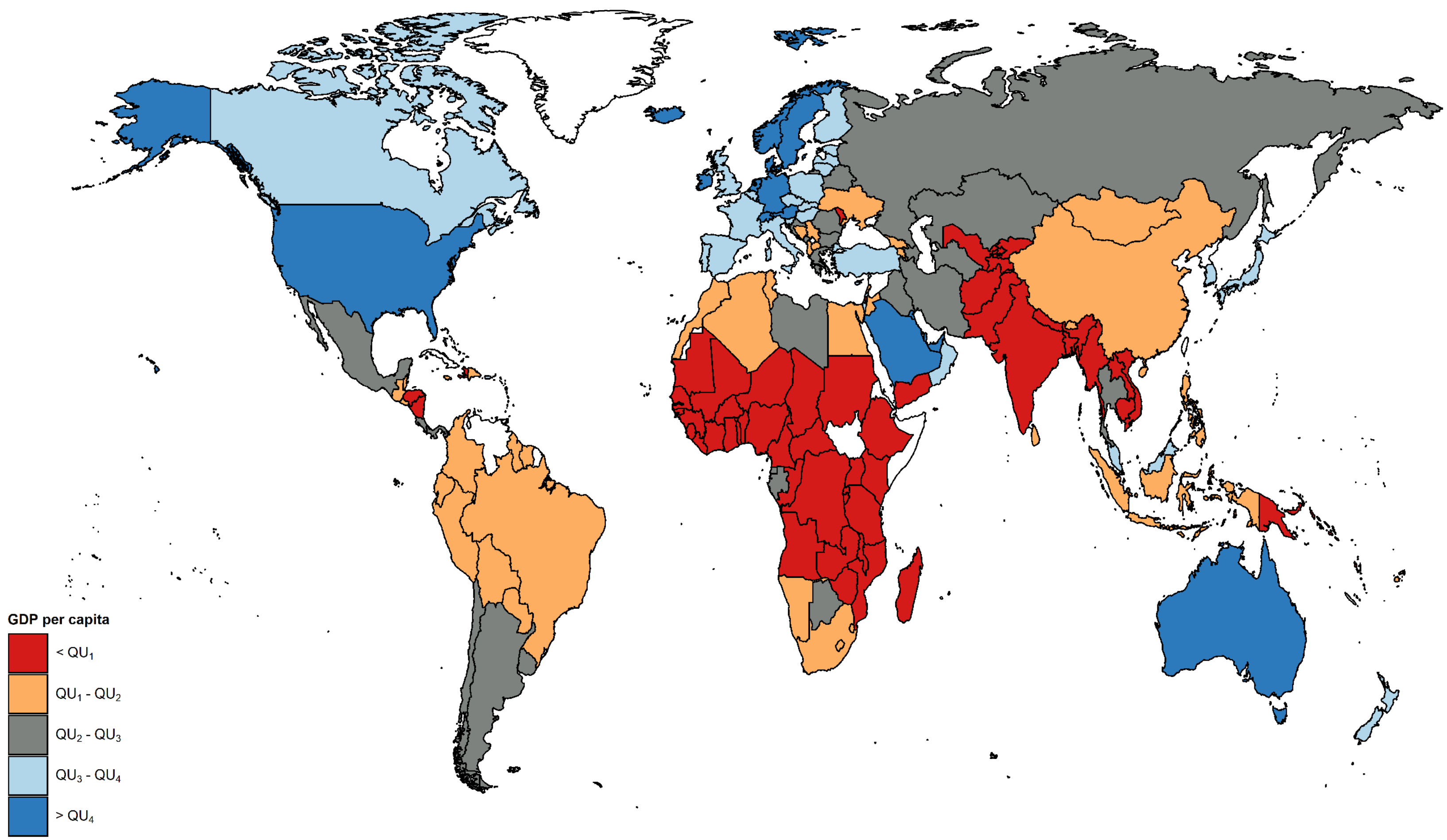

Figure A3. Map of per capita GDP in 2017 expressed in quintiles (QU).

Table A2. List of countries.

Afghanistan	Albania	Algeria
American Samoa*	Andorra#	Angola
Antigua and Barbuda	Argentina	Armenia
Australia#	Austria#	Azerbaijan
Bahamas	Bahrain	Bangladesh
Barbados	Belarus	Belgium#
Belize	Benin	Bermuda*
Bhutan	Bolivia	Bosnia and Herzegovina
Botswana	Brazil	Brunei
Bulgaria	Burkina Faso	Burundi
Cambodia	Cameroon	Canada#
Cape Verde	Central African Republic	Chad
Chile	China	Colombia
Comoros	Congo-Brazzaville	Congo-Kinshasa
Costa Rica	Croatia#	Cuba
Cyprus#	Czechia#	Denmark#
Djibouti	Dominica	Dominican Republic
Ecuador	Egypt	El Salvador
Equatorial Guinea	Eritrea	Estonia
Ethiopia	Fiji	Finland#
France	Gabon	The Gambia
Georgia	Germany#	Ghana
Greece	Greenland*#	Grenada
Guam*	Guatemala	Guinea
Guinea-Bissau	Guyana	Haiti
Honduras	Hungary	Iceland#
India	Indonesia	Iran
Iraq	Ireland#	Israel
Italy#	Ivory Coast	Jamaica
Japan	Jordan	Kazakhstan
Kenya	Kiribati	Kuwait
Kyrgyzstan	Laos	Latvia
Lebanon	Lesotho	Liberia
Libya	Lithuania#	Luxembourg#
Madagascar	Malawi	Malaysia
Maldives	Mali	Malta#
Marshall Islands	Mauritania	Mauritius
Mexico	Micronesia	Moldova
Mongolia	Montenegro	Morocco
Mozambique	Myanmar	Namibia
Nepal	Netherlands#	New Zealand#
Nicaragua	Niger	Nigeria
North Korea	North Macedonia	Northern Mariana Islands*
Norway#	Oman	Pakistan
Palestine	Panama	Papua New Guinea

Paraguay	Peru	Philippines
Poland	Portugal	Puerto Rico*
Qatar	Romania	Russia
Rwanda	Saint Lucia	Saint Vincent and Grenadines
Samoa	Sao Tome and Principe	Saudi Arabia
Senegal	Serbia	Seychelles
Sierra Leone	Singapore	Slovakia
Slovenia#	Solomon Islands	Somalia
South Africa	South Korea	South Sudan*
Spain#	Sri Lanka	Sudan
Suriname	Swaziland	Sweden#
Switzerland	Syria	Taiwan#
Tajikistan	Tanzania	Thailand
Timor-Leste	Togo	Tonga
Trinidad and Tobago	Tunisia	Turkey
Turkmenistan	Uganda	Ukraine
United Arab Emirates	United Kingdom	United States#
Uruguay	US Virgin Islands*	Uzbekistan
Vanuatu	Venezuela	Vietnam
Yemen	Zambia	Zimbabwe

Notes: n = 195. * Countries not present in GSQ. # Developed countries in terms of the burden of hunger. Missing countries (except island and city-states) include Kosovo, French Guiana and Western Sahara.

Table A3. Definitions of variables and data sources that differ from GSQ.

Variable name	Definition	Difference with GSQ	Source
Burden of hunger			
DALYs for various hunger-related cause factors	DALYs lost due to hunger-related diseases or injuries (that is, nutritional deficiencies) that directly cause death or disability	Updated methodology and data. See GBD 2017 collaborators (2019, 2018) for details.	GBD 2017
DALYs for various hunger-related risk factors (per cause-risk attribution)	DALYs lost due to hunger-related factors which are causally associated with the probability of a disease or injury. A selection of relevant health outcomes is stated in GSQ Table S1.	Updated methodology and data. See GBD 2017 collaborators (2019, 2018) for details. Note that GSQ Table S1 refers to intestinal infectious diseases instead of enteric infections and FeD anaemia instead of dietary iron deficiency.	GBD 2017
Basic determinants			
GDP per capita (PPP, const. 2011 international $)	See GSQ	The reference year is 2011 instead of 2005	WDI
Mean temperature	Country's average annual temperature measured in Celsius. The variable was calculated based on monthly temperature data.	New variable	CCKP (calculated)
Market potential	Weighted sum of country-level GDP (current US$). Weights are the inverse great-circle-distance between the most important cities/agglomerations (in terms of population) concerning two countries. See Mayer and Zignago (2011) for details.	New variable	CEPII (calculated)
Trade openness	Ratio of exports (current US$) over country-level GDP (current US$).	New variable	IMF DOTS (exports)<br>WDI (GDP) (calculated)
ICRG risk index	ICRG composite financial, economic, and political risk rating	New variable	HU ICRG
Food supply			

Total cereal availability (kg/capita/year)	Sum of cereals production (kg/capita/year) and cereals import quantity (kg/capita/year)	New variable	FAO FB (calculated)
Supply of plant products (kcal/capita/day)	See GSQ	Referred to as vegetal products instead of plant products	FAO FB
Supply of roots and tubers (kcal/capita/day)	See GSQ	Referred to as starchy roots instead of roots and tubers	FAO FB
Health and gender			
Access to safe water (% of population)	People using at least basic drinking water services (% of population)	This indicator encompasses both people using basic water services as well as those using safely managed water services. Smaller sample size available.	WDI
Access to improved sanitation (% of population)	People using at least basic sanitation services (% of population)	This indicator encompasses both people using basic sanitation services as well as those using safely managed sanitation services. Smaller sample size available.	WDI
Other			
Population	Total population (both sexes combined) by region, subregion and country, annually for 1950-2100.	Not specified in GSQ	UN DESA
World bank regions	World regions as defined by the World Bank (with some extension for non-classified countries).	More complete list of countries.	Lenaerts (2020)
Country ISO codes	Conversion file to match different spellings of country names to (unique) ISO3 country codes.	More complete list of countries.	Lenaerts (2020)

Notes: The remaining variables and data sources can be found in GSQ Table 8. CCKP refers to data downloaded from the Climate Change Knowledge Portal (CCKP) published by the World Bank (World Bank, 2019a). CEPII refers to the Centre d'Etudes Prospectives et d'Informations Internationales (CEPII, 2019). IMF DOTS refers to the International Monetary Fund (IMF) Direction of Trade Statistics (DOTS) (IMF DOTS, 2018). FAO FB refers to data published by the Food and Agriculture Organization (FAO) statistics division (FAOSTAT) under the domain food balance (FB) (FAOSTAT, 2018). GBD 2017 refers to the Global Burden of

Diseases, Injuries, and Risk Factors Study 2017 (IHME, 2019). HU ICRG refers to the International Country Risk Guide (ICRG) Researchers from Harvard Dataverse at Harvard University (ICRG Researchers, 2013). UN DESA to the United Nations Department of Economic and Social Affairs (Population Division) (UN DESA, 2019). WB KGCC to data downloaded from the Köppen-Geiger Climate Classification (KPCC) published by the World Bank (World Bank, 2019b). WDI refers to data obtained from the World Development Indicators (WDI) (World Bank, 2019c).

Table A4 Replication of the GSQ model regressing the burden of chronic and hidden hunger on basic determinants with new panel data spanning 1990–2017.

	Burden of chronic hunger				Burden of hidden hunger			
	(1) Fixed effects	(2) Fixed effects	(3) Random effects	(4) Random effects	(1a) Fixed effects	(2a) Fixed effects	(3a) Random effects	(4a) Random effects
Log(GDP per capita, PPP)	−0.4065***	−0.3956***	−0.4257***	−0.5024***	−0.3301***	−0.3275***	−0.2995***	−0.3115***
	(0.1146)	(0.1081)	(0.0883)	(0.0805)	(0.0591)	(0.0578)	(0.0382)	(0.0349)
Urban population (per cent of total)	−0.0331***	−0.0327***	−0.0146***	−0.0145***	−0.0210***	−0.0209***	−0.0098***	−0.0084***
	(0.0077)	(0.0069)	(0.0053)	(0.0045)	(0.0038)	(0.0037)	(0.0024)	(0.0021)
Population aged 0–14 years (% of total)	0.1003***	0.0905***	0.1295***	0.1256***	0.0506***	0.0483***	0.0633***	0.0586***
	(0.0101)	(0.0099)	(0.0094)	(0.0083)	(0.0056)	(0.0056)	(0.0046)	(0.0040)
Electoral democracy (dummy=1)	−0.1719**	−0.1617**	−0.0953	−0.1381*	−0.0895*	−0.0872*	−0.1023**	−0.1219***
	(0.0802)	(0.0798)	(0.0809)	(0.0750)	(0.0472)	(0.0459)	(0.0450)	(0.0423)
Average rainfall (mm)	−0.0020	−0.0018		−0.0005	−0.0010	−0.0010		−0.0012***
	(0.0014)	(0.0014)		(0.0009)	(0.0006)	(0.0006)		(0.0004)
Average temperature (Celsius)		−0.2075***		0.0462***		−0.0489**		0.0097**
		(0.0567)		(0.0099)		(0.0205)		(0.0044)
% land area in temperate zones			−0.6989**				−0.1065	
			(0.2822)				(0.1147)	
% land area in tropics and subtropics			0.6379***				−0.1183	
			(0.2167)				(0.1026)	
Number of observations	489	489	427	489	489	489	427	489
Number of countries	175	175	153	175	175	175	153	175
R-squared	0.71	0.74	0.84	0.82	0.77	0.78	0.85	0.84

Notes: These are results from country-fixed effects panel regressions spanning the years 1990, 2005 and 2010. Only countries included by GSQ are used here. One-way clustered standard errors at the country-level are shown in parentheses. The dependent variable is the logarithm of DALYs per capita lost due to chronic hunger (models 1–4) and hidden hunger (models 1a–4a). GDP, gross domestic product. PPP, purchasing power parity. ***, ** and * denote significance at the 1, 5 and 10 per cent level, respectively. These results are a replication of Table 2 in GSQ.

References

CEPII, 2019. <http://www.cepii.fr/CEPII/en>.

Croissant, Y., Millo, G., Tappe, K., Toomet, O., Kleiber, C., Zeileis, A., Henningsen, A., Andronic, L., Schoenfelder, N., 2019. plm: Linear Models for Panel Data. R package version 2.2-0.

FAOSTAT, 2018. <http://faostat.fao.org>.

GBD 2017 collaborators, 2019. Health effects of dietary risks in 195 countries, 1990–2017: a systematic analysis for the Global Burden of Disease Study 2017. The Lancet 393, 1958–1972.

GBD 2017 collaborators, 2018. Global, regional, and national disability-adjusted life-years (DALYs) for 359 diseases and injuries and healthy life expectancy (HALE) for 195 countries and territories, 1990–2017: a systematic analysis for the Global Burden of Disease Study 2017. The Lancet 392, 1859–1922.

Gödecke, T., Stein, A.J., Qaim, M., 2019. Corrigendum to 'The global burden of chronic and hidden hunger: Trends and determinants.' Global Food Security 22, 46.

Gödecke, T., Stein, A.J., Qaim, M., 2018. The global burden of chronic and hidden hunger: Trends and determinants. Global Food Security 17, 21–29.

ICRG Researchers, 2013. International Country Risk Guide (ICRG) Researchers Dataset. https://doi.org/10.7910/DVN/4YHTPU

IHME, 2019. Global Burden of Disease Study 2017 (GBD 2017) Results. Available from http://ghdx.healthdata.org/gbd-results-tool. IHME, Seattle, United States.

IHME, 2013a. GBD 2010: Results by cause 1990–2010. Available from http:// ghdx.healthdata.org/record/global-burden-disease-study-2010-gbd-2010-results-cause-1990-2010-country-level. IHME, Seattle, United States.

IHME, 2013b. GBD 2010: Results by risk factor 1990–2010. Available from http://ghdx.healthdata.org/record/global-burden-disease-study-2010-gbd-2010-results-risk-factor-1990-2010. IHME, Seattle, United States.

IMF DOTS, 2018. <http://data.imf.org>.

Leifeld, P., 2017. texreg: Conversion of R Regression Output to LaTeX or HTML Tables. R package version 1.36.23.

Leifeld, P., 2013. texreg: Conversion of Statistical Model Output in R to LATEX and HTML Tables. Journal of Statistical Software 55, 1–24.

Lenaerts, B., 2020. <https://github.com/BertLenaerts/geodata.git>.

Mayer, T., Zignago, S., 2011. Notes on CEPII's distances measures: The GeoDist database. CEPII.

R Core Team, 2020. R: A language and environment for statistical computing. R Foundation for Statistical Computing. Vienna, Austria.

UN DESA, 2019. <https://population.un.org/wpp/Download/Standard/Population>.

Wickham, H., 2020. tidyverse: Easily Install and Load the "Tidyverse". R package version 1.2.1.

World Bank, 2019a. <https://climateknowledgeportal.worldbank.org/download-data>.

World Bank, 2019b. <https://datacatalog.worldbank.org/dataset/world-maps-k%C3%B6ppen-geiger-climate-classification>.

World Bank, 2019c. <https://data.worldbank.org>.
